# Work stress in nurses returning to tertiary a general hospitals in China after the delivery of their second child: a cross-sectional study

**DOI:** 10.1186/s12913-022-07912-8

**Published:** 2022-04-13

**Authors:** Kai Chen, Lili Wei, Yan Zhang, Wenbin Jiang, Jingyuan Wang, Yueshuai Pan

**Affiliations:** 1 Department of Critical Care Medicine, The Affiliated Hospital of Qingdao University, Qingdao, China; 2 Department of Nursing, The Affiliated Hospital of Qingdao University, 16 Jiangsu Road, Qingdao, 266003 China; 3 Department of Respiratory and Critical Care Medicine, The Affiliated Hospital of Qingdao University, Qingdao, China

**Keywords:** Return to work, Nurses, Occupational stress, Cross-sectional studies

## Abstract

**Objective:**

To investigate the current situation of work stress in nurses returning to work in Chinese tertiary A general hospitals after giving birth to their second child and to analyze influencing factors.

**Methods:**

From January to April 2021, 448 nurses returning to work after the birth of their second child, working in 23 general hospitals in China, were investigated and completed the postpartum work stress scale and self-rating depression scale.

**Results:**

The total work stress score of returning nurses after giving birth to their second child was 90.40 ± 18.29, and the dimension with the highest score was the role commitment of the mother. Multiple linear regression analysis showed that family monthly income, turnover intention, time since returning to work, age of the first child, and depressive symptoms were the influencing factors on work stress.

**Conclusion:**

It is important to reduce the work stress of the nurses returning to work after the birth of their second child. Nursing managers should pay attention to this group of postpartum nurses and formulate targeted measures to alleviate their work stress.

**Supplementary Information:**

The online version contains supplementary material available at 10.1186/s12913-022-07912-8.

## Introduction

The nursing profession is a high-risk occupation accompanied by high work stress and physical and mental exhaustion. Nurses are faced with a large number of stressors at any time [1]. Throughout the world, the nursing profession is still dominated by women [2, 3]. By the end of 2019, the number of registered nurses in China had reached 4.43 million [4], within which the proportion of women was 97.7%. The age profile was younger than that of many other occupations, with 60.3% being under 35 years old, and therefore of childbearing age [5].

Female nurses have higher work stress compared to men [6]. Most of their stress comes from family and partner dependence [7]. At the same time, many nurses have increased work-family conflicts due to changes in family structure and distractions after having children, which can cause more stress for nurses with children [8, 9].

China adjusted its national fertility policy in 2016, changing the concept of only one child for a couple proposed in the 1980s to being able to have two children [10]. As a result, nursing, dominated by women of childbearing age, ushered in the climax of second childbirth. At present, the duration of maternity leave in China ranges from 98 days to 1 year, according to regional differences, and most women need to return to work after taking statutory maternity leave.

Due to the particularity of nursing work, the problems faced by nurses returning to work following the birth of their second child are very complicated. Firstly, 1 year after giving birth women are still in a transitional period in their physiology and psychology. In addition to continuing to experience physical health problems caused by childbirth [11], many women are prone to depression, negativity, anxiety, and other negative emotions due to the decrease and fluctuation of hormone levels such as estrogen, oxytocin, and prolactin in the perinatal period [12]. Secondly, the tertiary A general hospital is the highest level in the classification of medical institutions in mainland China. They are the medical institutions with strong comprehensive strength in clinical, scientific research, teaching and management, and they are the mainstay of the medical service system. The workload in a tertiary A general hospital is heavy, the work is difficult, the medical risk is great [13], the continuity of postpartum nurses’ work is interrupted after their long maternity leave, and the problem of poor work adaptation generally occurs after they return to work [14, 15]. Finally, the postnatal return period coincides with the breastfeeding period, and after returning to work these nurses also need to face the double stress of family care and the upbringing of two children. This further increases the conflict between work and family [16]. As a result, nurses returning to work after giving birth to their second child face higher work stress than before giving birth [17]. Therefore, how to effectively relieve the work stress of nurses returning to work after giving birth, and to strengthen their physical and mental health, has become a key issue requiring attention.

At present, there are few studies on postpartum nurses returning to work, and the current situation and influencing factors on the work stress of nurses with two children are not clear. Based on this, the purpose of this study is to investigate work stress in nurses returning to work in Chinese tertiary A general hospitals after giving birth, and to analyze the influencing factors. The study will provide a reference base for hospital managers to take targeted management measures to alleviate the work stress of postpartum returning nurses and to improve the quality of nursing.

## Objectives and methods

### Research objective

From January to April 2021, a convenience sampling method was used to select nurses who had returned to work after giving birth to their second child. To ensure the comprehensiveness and representativeness of the study results, participants were selected from different geographic regions of China, a total of 23 Chinese Grade A general hospitals in 10 provinces and municipalities directly under the Central Government.

Inclusion criteria: (1) registered nurses in tertiary A general hospitals; (2) returning to work within 1 year after their second maternity leave.

Exclusion criteria: (1) after returning to the post, they asked for leave again for more than 1 month due to their own illness; (2) they were clearly diagnosed with depression before or after delivery.

### Research tools

Three research tools were used in this study: a general information questionnaire, a postnatal return-to-work stress scale, and a self-rating depression scale.

#### General information questionnaire

The general information questionnaire was designed by the researchers on the basis of a literature review and included the age, educational background, mode of appointment (continuing/permanent appointment or fixed-term contract), department, family income, birth order, children’s sex, and length of maternity leave.

#### The work stress scale

Participants’ present level of work stress was measured by the work stress scale developed by our research team [18]. Guided by Cognitive Phenomenological Transactional (CPT) theory, the scale adopted the methods of literature review, semi-structured interviews, and group discussions to form the item pool. A total of 24 experts in nursing management, clinical nursing, and psychology were consulted from 10 tertiary A general hospitals in 8 provinces. The items of the scale were adjusted and screened using the Delphi method, and by item analysis and exploratory factor analysis. The final scale consists of a total of 30 items reflecting five dimensions: nursing work, family and work conflict, interpersonal relationship, patient nursing, and maternal role commitment. All items were scored on a five point Likert scale, and the scores ranged from one to five according to the frequency of “never,” “rarely,” “sometimes,” “often,” and “always”. The higher the score, the greater the work stress. The reliability and validity of the scale has been tested among the postpartum returning nurses in China’s Tertiary A general hospitals. The investigation of 635 postpartum nurses showed that the Cronbach’s α coefficient of the total scale was 0.94, and the Cronbach’s α coefficient of each dimension was 0.73–0.89. The content validity was 0.81–1.00, the confirmatory factor analysis showed that the five-factor model fitted well (*χ*^2^/df = 2.92, RMSEA = 0.06, GFI = 0.89, NFI = 0.89, IFI = 0.93, TLI = 0.92, CFI = 0.93), and the content validity was 0.81–1.00. An additional file shows the specific contents of this scale in more detail [see Additional file 1].

#### Self-rating depression scale

A Self-rating Depression Scale (SDS) [19] was used to evaluate the depression level of participants. It has been widely used in China, and studies have shown that it has good reliability and validity in China, while it is easy to use and can accurately reflect the depressive symptoms of the investigators [20]. The scale included 20 items and was scored using a four point Likert scale. The frequency of symptoms was rated as “never or rarely,” “occasionally,” “frequently,” and “most of the time/always” with one to four points respectively. It contained 10 reverse scoring entries, and the integer part of the total score multiplied by 1.25 was the standard score. According to the results of the Chinese norm, the SDS scored 53–62 points as mild depression, 63–71 points as moderate depression, and 72 points or more as severe depression [21]. In this survey, the Cronbach’s α coefficient of the scale is 0.839.

### Data collection

Because the subjects were distributed all over the country, the researchers made all the scales into electronic questionnaires so that they could be answered anonymously online. The participants clicked on the link or scanned the QR code on their computer or mobile phone terminal to fill in the electronic questionnaires. After completing the questionnaires, they uploaded the results directly.

Before the investigation, the researchers contacted the heads of the nursing departments in the hospitals concerned to introduce the contents and cooperation methods of this study and to screen for participants who met the exclusion criteria. After obtaining the informed consent of each participant, researchers sent them a link to the questionnaires. The introduction to the questionnaires explained in detail the purpose, significance and content of the study, and emphasized the confidentiality of participants’ data and responses. Submit all questions after filling in, and there were no limits placed on the time and place of answering the questions, but each subject was only allowed to answer once.

After the questionnaires were collected, in order to ensure their validity and the authenticity and integrity of the data, we used double verification to delete abnormal questionnaires in which participants’ answers were obviously regular or illogical.

### Statistical method

SPSS 22.0 software was used for the statistical analysis. The counting data were described by frequency and constituent ratio, and the measurement data were described by mean ± standard deviation. Where relevant the means of two groups were compared using independent sample t-tests and the means of multiple groups were compared using factor analysis of variance; and the pairwise comparison was analyzed using a Least Significant Difference test with multiple linear regression. Statistical significance was set at *P* < 0.05.

### Ethical consideration

This study was approved by the Ethics Committee of the affiliated hospital of Qingdao University (Ethics approval part number: QYFY WZLL 25658). The researchers guaranteed to provide participants with an explanation of the purpose of the study and to abide by the principles of anonymity and confidentiality. In addition, when nurses with depression were identified during the survey, although they could not be included in the study according to the exclusion criteria, the researcher would advise them to seek professional psychological counseling to help them avoid further worsening of their depression.

## Results

### Work stress scores of nurses returning to work after the birth of their second child

The convenience sampling method was used to select nurses who returned to work after giving birth. Participants were selected from 23 tertiary A general hospitals in 10 Chinese provinces and municipalities directly under the Central Government. A total of 497 questionnaires were collected 448 of which were valid, an effective recovery rate of 90.14%. The total work stress score of participants returning to work after giving birth was (90.40 ± 18.29), and the average Likert score of items was (3.01 ± 1.13). Among all dimensions, the score of “mother’s role commitment” was the highest, followed by “nursing work.” The total score and the scores of each dimension are shown in Table 1.

**Table 1:** Scores of each dimension of work stress of nurses returning to work after giving birth to the second child (*n* = 448)

Dimension	Score($\overline x$ ± s)	Equal distribution of entries($\overline x$ ± s)	Scoring rate(%)
Assume the role of mother	15.31 ± 3.01	3.83 ± 1.00	76.64
Nursing work	27.06 ± 6.34	3.38 ± 1.09	67.66
Patient care	13.99 ± 3.22	2.80 ± 0.95	55.97
Family and work conflict	18.46 ± 5.42	2.64 ± 1.04	52.76
Interpersonal relationship	15.57 ± 4.53	2.60 ± 1.07	51.59
Total score	90.40 ± 18.29	3.01 ± 1.13	60.27

### Comparison of work stress scores participants with general data and different characteristics

Among the 448 subjects, the childbearing age was 26–43 (33.72 ± 3.46) years, the age of the first child was 1–17 (5.82 ± 3.36), 244 (54.46%) were nurses, and 272 (60.71%) were screened positive for depression. The results of t-test and univariate analysis showed that monthly family income, time of maternity leave, time of returning to work, turnover intention, and level of depression all influenced participants’ work stress score, and the difference was statistically significant (*P* < 0.05). Further pairwise analysis showed that the participants with a family monthly income < 10,000 yuan, maternity leave > 158 days, return to work time < 1 month and 2–3 months, and with moderate or severe depression had higher work stress scores. The specific results are shown in Table 2.

**Table 2:** Comparison of work stress scores of postpartum nurses with different characteristics

Project	Number of people (%)	Score	F/t value	*P* value
Age (years)			1.318^a^	0.177
26–30	86(19.20)	90.79 ± 19.45		
31–35	228(50.89)	91.21 ± 18.18		
≥ 36	134(29.91)	88.76 ± 17.57		
Degree			0.870^a^	0.420
Junior college and below	53(11.83)	92.45 ± 20.64		
Undergraduate course	381(85.04)	90.30 ± 18.06		
Master’s degree or above	14(3.13)	85.36 ± 15.97		
Professional title			1.028^a^	0.359
Junior Nurse	20(4.46)	92.80 ± 20.85		
Senior Nurse	244(54.46)	89.29 ± 19.09		
Chief nurse or above	184(41.07)	91.61 ± 16.13		
Section			1.390^a^	0.237
Internal Medicine	138(30.80)	92.61 ± 17.78		
Surgery	99(22.10)	90.12 ± 18.50		
Department of Obstetrics and Gynecology	98(21.88)	88.21 ± 19.83		
Acute and critical illness	54(12.05)	92.39 ± 17.04		
Other departments	59(13.17)	87.51 ± 16.57		
Mode of appointment			0.328^b^	0.743
Nurse with fixed-term contract	285(63.62)	90.61 ± 19.63		
Nurse with continuing/permanent appointment	163(36.38)	90.02 ± 16.29		
Monthly household income			5.652^a^	0.004
< 10,000 yuan	171(38.17)	93.91 ± 19.54		
10,000 to 20,000 yuan	220(49.11)	88.77 ± 16.76		
> 20,000 yuan	57(12.72)	86.16 ± 18.24		
Is there any intention to leave?			9.502^b^	0.000
Yes	122(27.23)	102.66 ± 18.15		
No	326(72.77)	85.81 ± 16.09		
Maternity leave time (days)			3.895^a^	0.021
≤ 90	12(2.68)	80.25 ± 14.78		
91–158	371(82.81)	89.95 ± 18.32		
>158	65(14.51)	94.82 ± 17.56		
Return to work time			8.028^a^	0.000
< 1 month	26(5.80)	103.96 ± 19.55		
2–3 months	75(16.74)	101.64 ± 17.54		
4–6 months	140(31.25)	91.74 ± 17.83		
7–9 months	146(32.59)	84.19 ± 14.52		
10–12 months	61(13.62)	82.57 ± 17.00		
The mode of pregnancy of this birth			0.101^b^	0.920
Natural conception	445(99.33)	90.41 ± 18.34		
Assisted reproduction	3(0.67)	89.33 ± 3.86		
Fetal sex			-0.574^b^	0.566
Boy	260(58.04)	89.98 ± 17.90		
Girl	188(41.96)	90.98 ± 18.79		
Age of one child (years)			1.288^a^	0.288
1–5	257(57.37)	91.57 ± 19.41		
6–10	144(32.14)	88.97 ± 17.80		
≥ 11	47(10.49)	88.38 ± 13.28		
Depressive symptoms			39.487^a^	0.000
No	176(39.29)	82.42 ± 16.28		
Mild	154(34.38)	90.75 ± 16.91		
Moderate	87(19.42)	98.00 ± 14.94		
Heavy	31(6.92)	112.61 ± 16.23		

^a^F value

^b^t value

### Analysis of multiple factors influencing work stress of postpartum nurses returning to work

Participants’ total work stress score was taken as the dependent variable. The general data and participants’ self-rating depression scale were divided into independent variables, a multiple linear regression analysis was carried out, and the data were entered. Before the analysis, the dummy variables such as education, professional title, department, and other classification variables were set, and the continuous numerical data such as age, maternity leave time, return to work time, and the age of the first child were directly entered and analyzed. The results showed that family monthly income, turnover intention, time of returning to work, age of the first child, and depression were the factors influencing participants’ work stress. The specific results are shown in Table 3.

**Table 3:** Multivariate linear regression analysis of the influencing factors of work stress of nurses returning to work after the second child

Variables	Unstandardized coefficients (B)	Std. error (SE)	Standardized coefficients(β)	t	*P*
Constant	119.146	12.232	–	9.741	0.000
Monthly household income	−2.062	1.017	−0.086	−2.028	0.043
Turnover intention	8.445	1.904	0.206	4.435	0.000
Return to work time	−5.019	0.681	−0.295	−7.367	0.000
The age of the first child	−0.646	0.310	− 0.121	−2.081	0.038
Depressive symptoms	−5.503	1.502	−0.150	−3.664	0.000

R = 0.610, adjusted R^2^ = 0.334, F = 9.615, *P* = 0.000

## Discussion

The postpartum returning nurses should be a key concern group for nursing managers. The increased stress of returning to work after maternity leave will lead to serious tension and difficulty in concentration, which will seriously affect their physical and mental health [22], and also lead to postpartum returning nurses becoming a potential high-risk group for clinical nursing errors and accidents, which will reduce the quality of nursing care [23]. In previous studies, the research tools used to investigate the stress of postpartum returning nurses have been generic work stress scales. This study is the first to use a specific work stress assessment tool for postpartum returning nurses. The results show that the dimension with the highest score in this study was “maternal role commitment.” This contrasts with previous research, in China and other countries, in which “nursing profession and work,” has been identified as the most significant stressor for clinical nurses [24, 25]. The main stressors were “breastfeeding time cannot be guaranteed after returning to work” and “lack of physical strength and energy due to childcare after returning to work.”

There are several reasons for this difference. Firstly, although nurses can temporarily leave children in the care of grandparents when they are working, according to Chinese tradition women need to take more care of their families. According to the surveys, in China, mothers are the primary caregivers of infants and toddlers aged 0–3 years [26], and the percentage of children who are primarily cared for by their mothers at night is as high as 74.1% [27]. Therefore, the vast majority of postpartum returning nurses still need to care for their children after work. This leads to nurses’ need to combine more maternal roles and tasks with their return to work and to invest more time and energy in raising their children.

Secondly, the nurses with two children are faced with a greater problem of physical recovery because of age-related decline in their bodies or to the effects of birth and childcare [28]. The duration of maternity leave in China is 98 days, but each province has its own regulations. Most maternity leave is 98 to 158 days, and very few will be extended to 1 year. But in practice, some nurses are not entitled to full maternity leave due to the shortage of nurses in the department and the need to work [29]. As a result, many nurses not recovered to the physical demands of being able to stand for long periods of time and perform intense nursing work, and at the same time, they need to face the contradiction between taking care of their children and their own lack of physical strength and energy [30].

Finally, participants’ return to work coincides with their period of breastfeeding. The World Health Organization (WHO) recommends that babies be exclusively breastfed for the first 6 months and that breastfeeding should then continue for 2 years or more [31]. Chen’s [32] research points out that nurses’ period of breastfeeding in China is far from meeting the WHO recommendations, and is often actively interrupted by physical fatigue, increased work stress, and a lack of support in their departments. Although nurses have returned to work, as breastfeeding mothers they still need to express milk regularly during working hours. However, due to the continuity of nursing work, many departments cannot guarantee them the necessary time for this. In addition, in China there are also some deficiencies in the provision of suitable spaces in which nurses can express milk. In a survey of four tertiary A hospitals in Shandong Province, 64.25% of the departments did not have clean and private space in which women could express their milk, and 46.93% of the departments did not provide refrigerators, freezers, and other milk storage facilities [33]. These deficiencies increased nurses’ worries about breastfeeding after returning to work, further increasing their stress.

### The relationship between returning nurses’ stress and their level of family income

In 2020, the annual per capita disposable income of urban residents in China was 43,834 yuan, and the per capita consumption expenditure was 27,007 yuan. Although the income level of nurses in China is higher than the national average, it is not a highly-paid occupation. In particular, second-child nurses face the double stress of the material and parenting of two children, which increases the economic burden on the family [34]. In this study, 38.17% of nurses had a monthly income of less than 10,000 yuan, and the lower the family income, the higher their work stress. Cohen’s [7] research also confirms this. Many developed countries have relatively good maternity insurance systems, the welfare benefits given by their governments to nurses who are mothers are relatively generous, and various subsidies are provided for families to reduce their financial burden [35, 36]. Although China has formulated corresponding regulations on the protection of maternity leave, so that nurses can enjoy maternity leave allowance, maternity medical allowance and other benefits in accordance with the law, compared with developed countries there is still a big gap [37]. This increases the work stress of postpartum nurses from low-income families. China also needs to further improve the social welfare system to ensure that nurses are given appropriate incentives and benefits according to the actual situation of their local hospitals, while enjoying the maternity leave stipulated by the national policy. This would reduce the family burden on lower income groups.

### Nurses with younger first child have higher work stress

Shu’s [16] research points out that nurses returning to work following the birth of a second child need more time to take care of their older children’s needs and education, in addition to caring for their second child. This further adds to the demands made by their work. The results of this study show that the work stress of nurses with lower age of the first child was higher than child with older age. This may be because younger children, in the early childhood and preschool stage, are more active and curious, factors which contribute to this age group showing the highest incidence of accidental injuries [38]. At the same time, this is also an important period for children’s intellectual and social development. The formation of lifelong habits is completed at this stage [39, 40]. After a second birth, nurses have to face the heavy work and upbringing of two children and the time spent on the care of the first child is inevitably reduced. Therefore, managers should fully understand the family situation of second-child nurses, arrange shifts reasonably to help them better alleviate the conflict between work and family, and the nurses should encourage their husbands to actively assume family responsibilities and shoulder the task of taking care of their eldest children. This would help to create a good family atmosphere.

### The relationship between work stress in postpartum returning nurses and the passage of time

The results of this study show that participants’ levels of post-return work stress change over time, being higher in the first 3 months, especially in the first month, and show a downward trend with the extension of return time. This is similar to the results of studies on work stress and adaptation of postpartum returning nurses by Lin [41] and Chen [30]. This trend may be related to the fact that in such a short time after returning to their post, nurses with a second child find it difficult to adapt the high-intensity work rhythm, or master the changes in technology and knowledge that have occurred in their absence. However, with the passage of time, they can gradually become familiar with the necessary knowledge and skills, so as to reduce their level of work stress. Some researchers have pointed out that nurses with a second child have a low level of adaptation to work within 3 months after returning [42] and show a greater lack of self-confidence in completing their work, causing serious mental stress [43]. Therefore, it is suggested that nursing managers should pay special attention to the return of second-child nurses during the first 3 months after maternity leave. They should actively help these nurses to formulate a suitable post-return training plan and establish good family support relationships. Managers should dynamically adjust the nurses’ work according to their actual situation and according to the principle of gradual and orderly progress, so as to shorten the maladjustment period of returning to work and gradually reduce the nurses’ work stress.

### The relationship between depressive symptoms and work stress in postpartum returning nurses

Postpartum hormone changes in the body make women more prone to depression, anxiety, and other negative emotions. Kamau’s [44] research shows that postpartum depressive symptoms can seriously affect postpartum work status and weaken women’s ability to return to normal work. This study found that 60.71% of the participants who returned to work after giving birth had depressive symptoms, and the higher the level of depressive symptoms, the greater their work stress. A previous study [45] has found that depression scores are positively correlated with all dimensions of work stress scores. When nurses’ depressive symptoms cannot be addressed effectively, it will increase their work stress. Lin [46] has shown that nurses’ work stress is positively correlated with their level of depressive symptoms, and high work stress can also lead to severe depressive symptoms. Therefore, for pregnant nurses it is necessary to screen their state of prenatal and postpartum depressive symptoms, and to provide suitable support measures in advance, for example, by increasing mental health services and enhancing mental support. For postpartum nurses with a tendency towards depression, nursing managers can appropriately extend their maternity leave. In the absence of a long maternity leave, nursing managers should be aware of the possibility of postpartum depressive symptoms and provide appropriate support for postpartum returning nurses.

#### The relationship between work stress and turnover intention in postpartum returning nurses

In this study, 27.23% of nurses returned to work after giving birth to their second child, which is lower than the level of turnover intention found by Yang et al [47] in nurses across China. Studies [48] have shown that difficulties in balancing work and family roles lead to higher turnover intentions. Nurses returning to work after having a second child are faced with the heavy workload involved in caring for their family and children at the same time, and the conflict experienced between family and work commitments is serious. However, raising a family is expensive, many nurses cannot bear the economic losses caused by leaving their work. Even if they wanted to leave, they could not do so easily. Lai’s [49] research also confirms this. Therefore, many nurses who return to work after delivery want to leave, but have to continue to work, which undoubtedly increases their work stress. Managers should pay attention to the turnover intention of postpartum returning nurses, and take a scientific and human approach to their management that meets their life and psychological needs. Nurses themselves should actively try to maintain good mental health and strengthen their ability to adjust to their new circumstances.

This study gives the researcher food for thought in terms of gender inequality. Looking at the global health industry, women typically receive fewer opportunities for advancement and lower salaries than men, and the impact of gender differences adds to the work stress of female health care workers [50, 51]. Women face the same competition as men in the labor market and are required to meet the same work standards as men, but in the home, women take on more responsibility for caring for their families. Therefore, managers at all levels should pay attention to the realization and protection of women’s rights and interests, promote the trend toward equal social resources owned by both genders, change gender equality within the family, and help nurses returning to work after childbirth to share the task of child care in order to help them reduce stress.

## Limitations of the study

1. Although hospitals were selected from different regions across China to ensure a wide range of samples, the sampling in this study was non-randomized and the sample size in each hospital and region was different. There is no effective comparative analysis on the work stress of nurses returning to work after giving birth in different regions of China and there is a certain sample selection bias.
2. The depressive symptoms investigated in this study is not the same as postpartum depression. The researchers investigated participants’ depressive symptoms after returning to work, but they did not conduct a follow-up survey through the prenatal to postpartum periods and so did not have a comprehensive grasp of nurses’ postpartum depression as distinct from their work-related depression. On the other hand, the exclusion of those who have “diagnosed depression” can generate a limitation in the interpretation of the results.

## Summary

The postnatal return period is a challenging time that every pregnant nurse will experience. With the introduction of the two-child policy in China, second-child nurses face higher work stress during this period. This study indicates that nursing managers should actively intervene, adopting humanized management and formulating targeted measures to reduce the workload of postpartum nurses. These nurses also need the joint efforts of relevant social departments and hospital management departments to establish a postnatal return support system that alleviates their stress and ensures the stable development of the nursing team.

## Supplementary Information

**Additional file 1.**

## Data Availability

The data can be obtained from the corresponding author upon reasonable request.
