# Supplementary material for: Work stress in nurses returning to tertiary a general hospitals in China after the delivery of their second child: a cross-sectional study
Source: BMC Health Serv Res. 2022 Apr 13;22:492. doi: 10.1186/s12913-022-07912-8 (PMC9006626; doi:10.1186/s12913-022-07912-8)
Supplement: Supplementary file 1 — Additional file 1. [file 12913_2022_7912_MOESM1_ESM.docx]

**The work stress scale for the postpartum nurses who returned work**

Please read each item in the chart below carefully and mark "√" after the corresponding entry according to the frequency score below. There are no right or wrong answers below, please fill in your true feelings as much as possible.

| Dimensions | Items | Never | Rarely | Sometimes | Often | Always |
| --- | --- | --- | --- | --- | --- | --- |
| Maternal role commitment | Breastfeeding time cannot be guaranteed after returning to work | 1 | 2 | 3 | 4 | 5 |
|  | High financial investment of children and increased stress | 1 | 2 | 3 | 4 | 5 |
|  | Lack of physical strength and energy due to childcare after returning to work | 1 | 2 | 3 | 4 | 5 |
|  | Worrying about your child's diet and health at home while you are at work | 1 | 2 | 3 | 4 | 5 |
| Work-family conflict | Multiple family roles can interfere with work tasks | 1 | 2 | 3 | 4 | 5 |
|  | Family factors such as illness of family members distract from work | 1 | 2 | 3 | 4 | 5 |
|  | Easy to bring the emotions of family conflicts to work | 1 | 2 | 3 | 4 | 5 |
|  | Inability to adapt to updates in clinical knowledge, systems, workflow, instruments, etc. after returning to work | 1 | 2 | 3 | 4 | 5 |
|  | Inability to adapt to a stressful work environment after returning to work | 1 | 2 | 3 | 4 | 5 |
|  | Negative emotions in nursing affect the family atmosphere | 1 | 2 | 3 | 4 | 5 |
|  | Family members are less supportive of your work | 1 | 2 | 3 | 4 | 5 |
| Nursing work | Too much non-care work and useless paperwork | 1 | 2 | 3 | 4 | 5 |
|  | Too much training and exams outside of work hours | 1 | 2 | 3 | 4 | 5 |
|  | Too much workload | 1 | 2 | 3 | 4 | 5 |
|  | Low number of nurses on duty | 1 | 2 | 3 | 4 | 5 |
|  | Overcrowded wards | 1 | 2 | 3 | 4 | 5 |
|  | High stress from continuing education | 1 | 2 | 3 | 4 | 5 |
|  | Fear of infection, violence and other risks during work | 1 | 2 | 3 | 4 | 5 |
|  | Fear of errors and accidents at work | 1 | 2 | 3 | 4 | 5 |
| Patient nursing | Patients and families do not understand | 1 | 2 | 3 | 4 | 5 |
|  | High expectations of patients and families | 1 | 2 | 3 | 4 | 5 |
|  | Patient non-cooperation | 1 | 2 | 3 | 4 | 5 |
|  | Sudden death of a patient under care | 1 | 2 | 3 | 4 | 5 |
|  | Patients cared for are too sick | 1 | 2 | 3 | 4 | 5 |
| Interpersonal relationship | Lack of understanding and support among colleagues | 1 | 2 | 3 | 4 | 5 |
|  | Lack of friendly and cooperative atmosphere among colleagues | 1 | 2 | 3 | 4 | 5 |
|  | Insufficient understanding and support from nursing managers | 1 | 2 | 3 | 4 | 5 |
|  | Too much criticism from nursing managers | 1 | 2 | 3 | 4 | 5 |
|  | Fear of impact on co-worker relationships due to prolonged absence from work or deterioration in work quality | 1 | 2 | 3 | 4 | 5 |
|  | Small circle of life, little sense of communication with the outside world | 1 | 2 | 3 | 4 | 5 |
